# Artificial Intelligence to derive aligned strain in cine CMR to detect patients with myocardial fibrosis: an open and scrutinizable approach

**DOI:** 10.21203/rs.3.rs-3785677/v1

**Published:** 2024-01-05

**Authors:** Sven Koehler, Julian Kuhm, Tyler Huffaker, Daniel Young, Animesh Tandon, Florian André, Norbert Frey, Gerald Greil, Tarique Hussain, Sandy Engelhardt

**Affiliations:** 1 Department of Internal Medicine III, Heidelberg University Hospital, Heidelberg, Germany; 2 German Center for Cardiovascular Research (DZHK), Partnersites Heidelberg and Mannheim, Germany; 3 University Heidelberg, Heidelberg, Germany; 4 Division of Pediatric Cardiology, Department of Pediatrics, UT Southwestern /Children’s Health, 1935 Medical District Drive B3.09, Dallas, TX 75235, USA; 5 Department of Heart, Vascular, and Thoracic, Children’s Institute; Cleveland Clinic Children’s Center for Artificial Intelligence (C4AI); and Cardiovascular Innovation Research Center, Cleveland Clinic Children’s, Cleveland, Ohio, USA; 6 Department of Biomedical Engineering, Case School of Engineering, Case Western Reserve University, Cleveland, Ohio, USA

## Abstract

Cine Cardiac Magnetic Resonance (CMR) is the gold standard for cardiac function evaluation, incorporating ejection fraction (EF) and strain as vital indicators of abnormal deformation. Rare pathologies like Duchenne muscular dystrophies (DMD) are monitored with repeated late gadolinium-enhanced (LGE) CMR for identification of myocardial fibrosis. However, it is judicious to reduce repeated gadolinium exposure and rather employ strain analysis from cine CMR. This solution is limited so far since full strain curves are not comparable between individual cardiac cycles and current practice mainly neglects diastolic deformation patterns. Our novel Deep Learning-based approach derives strain values aligned by key frames throughout the cardiac cycle. In a reproducibility scenario (57+82 patients), our results reveal five times more significant differences (22 vs. 4) between patients with scar and without, enhancing scar detection by +30%, improving detection of patients with preserved EF by +61%, with an overall sensitivity/specificity of 82/81%.

## 1 Introduction

Duchenne muscular dystrophy (DMD) is an X-chromosomal linked and the most commonly inherited muscular disorder in childhood (incidence 1:3500 male newborns) [1]. It is caused by mutations in the dystrophin gene and, over time, induces a fibrofatty replacement of muscular tissue, ultimately leading to muscle weakness in all parts of the body [2]. With increasing age, cardiac involvement is almost universally present in DMD and manifests with dilated cardiomyopathy [3, 4], left-ventricular ejection fraction (LVEF) reduction [5, 6] and arrhythmia [4, 7]. As a result of improvements in respiratory care, cardiac failure is the leading cause of mortality in the DMD population [8, 9, 10]. During the follow-up of patients with DMD, Cardiac Magnetic Resonance (CMR) examinations including native series and Late Gadolinium Enhancement (LGE) are performed. They show an increased signal intensity for fibrotic myocardial regions [6, 11], which results from an expanded interstitial space where the contrast agent Gadolinium accumulates [12]. The serial assessment of ventricular volumetric and LGE has evolved as a standard prognostic tool to differentiate healthy (*LGE−*) and abnormal (*LGE+*) myocardium [6, 11] in patients without severely reduced renal function. That necessitates repeated gadolinium contrast injection, which includes an intravenous line and additional scanning time. Moreover, the exact quantification of LGE is cumbersome and requires a minimum fibrotic volume [6].

Thus, the development of alternative methods to detect myocardial fibrosis without contrast agent administration would be a considerable improvement. Previous research has highlighted the usefulness of cardiac strain from cine CMR as a predictor of abnormal myocardial deformation [13, 14, 15, 16, 17, 18, 19, 20, 21]. Those strain measurements are considered to be superior markers to asses the cardiac contractility compared to the LVEF measurements [22, 23, 24, 25].

**Figure 1:**
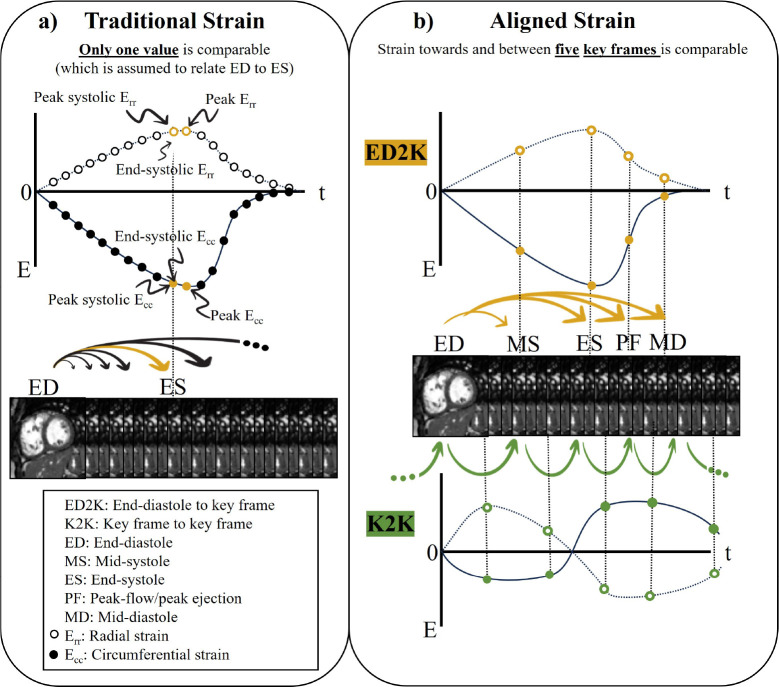
**a)**, Traditional strain measure. In this widely established workflow, each frame of an CMR sequence is compared to the end-diastolic (ED) key frame. This results in temporally highly-resolved strain curves. Unfortunately, due to varying number of frames and variable lengths of cardiac cycles and cardiac sub-phases, strain curves are not temporally aligned between patients and an automatic comparison of each strain value is not possible. For an inter-patient comparison either the peak strain, the peak systolic strain or the end-systolic strain is considered [28] with the goal of comparing maximal deformation of the myocardium during the systolic phase, however, this assumption may not hold in certain patient cohorts. **b)**, Proposed aligned strain measure through five key frames, derived from self-supervised contraction-relaxation curves [32]. This approach identifies five cardiac key frames throughout the cardiac cycle: end-diastole (ED, end of diastolic relaxation); mid-systole (MS, maximum systolic contraction); end-systole (ES, end of systolic contraction); peak flow (PF, peak early diastolic relaxation) and mid-diastole (MD, key frame before atrial contraction at p-wave onset). This enables to derive composed (*ED2K*) strain between the end-diastolic key frame and the other key frames and sequential, from one key frame to the next (*K2K*). Through this alignment, the temporal information of the whole cardiac cycle can be exploited and compared between different patients. Note the different strain curves and the higher sampling of key frames along the temporal axis. Dashed lines and empty circles denote radial strain $\left(E_{rr}\right)$, solid lines and dots depict circumferential strain $\left(E_{cc}\right)$. E: strain, t: time.

In general, myocardial deformation can vary in intensity, onset, duration, and location depending on the pathology. Deriving full radial $\left(E_{rr}\right)$ or circumferential $\left(E_{cc}\right)$ strain curves entails computing the myocardial deformation between the reference end diastolic (ED) key frame to all other frames (cf. Figure 1 a). While regional profiles can be obtained by dividing the myocardium in standardised segments [26], temporal alignment remains a major challenge but is pivotal to achieve inter-patient comparison. Semantic alignment of the derived full strain curves is not easily possible due to different temporal resolution and trigger times of the CMR, and varying duration of cardiac sub-phases where deformation occurs. It has been shown that the temporal resolution of the CMR series has a significant influence on the strain values [27].

The field has approached the problem by extracting a single strain value as descriptor of the systolic deformation [28, 24, 29]. One option is to select the maximum of the curve (peak strain) neglecting the positional encoding within the cardiac cycle and without further systolic isolation, it can potentially include post-systolic deformation [27]. A better positional alignment between patients can be achieved by determining the end-systolic frame (end systolic strain – strain between ED and ES frames) (e.g. [30, 13]) or deriving the peak strain within the systolic sub-phase (peak systolic strain between ED and ES frames). However, relying solely on systolic strain values significantly reduce the information on myocardial deformation. Sub-cohorts of patients with preserved LVEF, where the systolic phase itself may be a less predictive marker, could therefore fail in the analysis. Although calculating peak strain rates and diastolic strain rates (e.g. [30, 24, 31]) where the deformation is integrated over a temporal interval can pose a potential solution, a systematic investigation of non-handpicked phase intervals has remained poorly explored. Such an approach would require robust CMR-based extraction of several temporal markers (‘key frames’) along the cardiac cycle that goes beyond the current and error-prone LV volume calculations for ES and ED determination [27].

**Figure 2:**
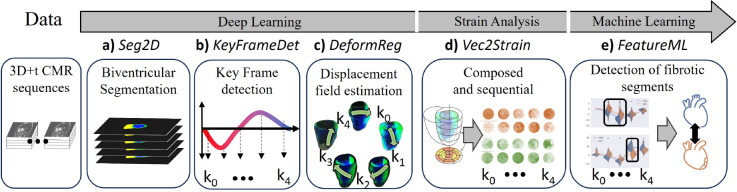
High-level description of the proposed pipeline. The proposed workflow consists of five components (cf. Section 5.3). First, steady-state free-precession cine images in short-axis view are provided. Then, myocardial tissues are segmented from a supervised model. In the following, multiple key frames throughout the cardiac cycle are detected using a self-supervised method from Koehler et. al [32]. Next, the short- and long-distance displacement fields between those key frames are learnt. In the fourth step radial $\left(E_{rr}\right)$ and circumferential $\left(E_{cc}\right)$ segment-wise strain is derived from the composed (*ED2K*) and sequential (*K2K*) displacement fields. Finally, the segment-wise and key frame specific strain values are used to predict patients with myocardial fibrosis.

We propose a method to derive aligned $E_{cc}$ and $E_{rr}$ cardiac strain from stacks of 2D steady-state free precession (SSFP) cine short-axis CMR to predict myocardial fibrosis in patients with DMD. For this purpose, a fully automatic Deep Learning and Machine Learning pipeline (cf. Figure 2 and 4) is introduced to derive composed (ED to key frames- *ED2K*) and sequential (key frame to key frame - *K2K*) strain values between five cardiac key frames that are distributed over the cardiac cycle (cf. Figure 1 b) and describe beginning and end of respective sub-phases. The key frames are extracted in a self-supervised manner (cf. Section 5.3) [32], meaning for learning this task no expert labels are required, and we show the robustness of the approach in several experiments (cf. Section 2). This constitutes 1) a novel variant to calculate strain, which allows for higher sampling along the temporal axis instead of taking only the two prominent key frames of ED and ES into consideration, which is the current state-of-the-art [27] (cf. Figure 1). 2) We rely on the calculation of strain tensors from dense displacement fields similarly as proposed by Morales et al. [30], which constitutes an alternative to feature tracking of segmented contours. 3) Our comprehensive analysis involves two cohorts comprising 139 (57+82) patients with the rare condition of DMD. One cohort is used in a cross-validation manner for model development and evaluation, while the other was introduced thereafter to systematically test the reproducibility of the approach on new clinical data.

## 2 Results

This work mainly compares the potential additional value of aligned composed (*ED2K*) and sequential (*K2K*) strain (cf. Figure 1 b). During development, robust architectures were extensively sought after by modifying three DL modules (cf. a–c in Figure 4 and in Section 5.3). The intermediate measures for each module of our final pipeline are reported briefly in Section 2.4 in favour of a more detailed evaluation regarding the potential value of aligned strain values (cf. Sections 2.2 and 2.3).

### 2.1 Study Cohorts

This study was approved by the institutional review board of the HRPP designated reviewers at UT Southwestern Medical Center, Dallas, Texas. The exempt criteria was met under 45 CFR 46.104(d). A coverage analysis was not required. A clinical trial agreement was not required.

Two single-center cohorts of male patients with DMD are investigated in our retrospective study. The patients in both cohorts underwent native CMR and LGE examinations (cf. Section 5.2). Based on the routine clinical reports cardiac segments are labelled as either LGE-positive (*LGE+*) or LGE-negative (LGE−). The first sub-cohort (DMD57) consists of 57 patients and is used in a four-fold cross validation manner for model development and provides additionally biventricular ground truth contouring at five distinct cardiac key frames together with the corresponding key frame indices (cf. Section 5.2). The images of this cohort have been acquired between 2018 and 2020. In this cohort, 33 patients have at least one myocardial segment with fibrosis *LGE+* (57.9%), on a segment level, 187 segments out of 912 are *LGE+* (20.5%).

A second retrospective cohort (DMD82) of 82 patients from the same center was acquired after model development to test the model’s reproducibility on new clinical data. The images were acquired at a later stage between February 2022 and April 2023. In this sub-cohort, no biventricular ground truth or cardiac key frames were labeled. Here, 51 patients (62.2%) and 266 segments (20.27%) are *LGE+*. Both cohorts were treated independently at all times. Table 1 lists the cohort-wise demographical parameters grouped by *LGE+*/*LGE*− and provides an overview of *p*-values within and between the two cohorts. There are no statistical significant differences in any of the parameters. Table 1 in the Supplemental Material summarises the MRI-related parameters.

Incorporating the involvement of the left ventricle ejection fraction (LVEF) as important clinical marker revealed an interesting picture. A LVEF threshold of = 55% was applied to further split patients with fibrosis (LGE+) into those with a preserved LVEF $\left({LGE+}_{pLVEF}\right)$ and those with a reduced LVEF $\left({LGE+}_{rLVEF}\right)$, similar as done by Earl et al. [33] (The first value in brackets refer to the *DMD57* cohort and the second to the *DMD82*): From the 139 patients, 84 patients were *LGE+* (33, 51) and 55 *LGE*− (24, 31). From the 84 *LGE+* patients 45 showed a reduced LVEF (19, 26) and 39 showed a preserved LVEF (14, 25). For the test cohort *DMD82* only half of the *LGE+* patients (26 of 51) developed a reduced LVEF. Interestingly, for the *LGE*− group (55) we found 10 patients (5, 5) with a reduced LVEF $\left({LGE+}_{rLVEF}\right)$.

**Figure 3:**
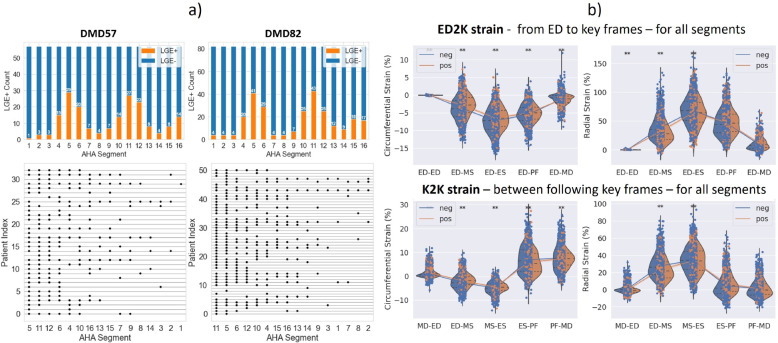
a) Distribution of *LGE+* AHA segments in the DMD cohorts, indicated by absolute numbers and dots. In both cohorts, the segments 4,5,6 and 10,11,12 were *LGE+* most frequently, followed by the segments 13,15,16. The stripplots provide a more detailed view of the *LGE+* cardiac segments in the patients of the two cohorts. In these plots, each line stands for a single patient, and dots indicate the AHA segment that was *LGE+*. Note that the axes of AHA segments are sorted in descending order of the segments’ *LGE+* occurrence. b) Mean circumferential ($E_{cc}$) and radial strain ($E_{rr}$) violinplots for all cardiac segments between the five cardiac key frames for the *ED2K* and *K2K* approach (DMD57+DMD82). Neg: *LGE*- (blue), Pos: *LGE+* (orange), asterisks indicate statistical significant differences without Holm-Bonferroni adjustment. Figure 1 and 2 in the Supplemental Material provide a detailed view split per segment.

Fig. 3 a) depicts the distribution of *LGE+* AHA segments in the two cohorts. In both cohorts, the basal inferior, inferolateral, and anterolateral (AHA4–6), mid inferior, inferolateral, anterolateral (AHA10–12) and apical inferior, lateral (AHA15–16) segments were the most affected. The stripplots reveal that in the DMD57 cohort, every *LGE+* patient had a positive segment in a combination of either AHA5,11,12 or AHA5,11,6. In the DMD82 sub-cohort, the occurrences are AHA5,11,12,14,15 or AHA5,11,12,14,16 to cover all *LGE+*. Note that the general pattern of affected segments seems robust across sub-cohorts. The segmental involvement align with the findings from related studies [34, 35].

### 2.2 Strain Analysis

The study sought to assess the handling of challenges in real clinical data, including extensive breathing and motion artifacts. The CMR series from both datasets underwent visual inspection. The following brackets list the number of series per quality group, the first number belongs to the *DMD57* and the second to the *DMD82* cohort. Images with no artifacts are categorised as good (20 and 29), series with moderate artifacts in some slices or frames are considered medium (30 and 40), and stacks with significant artifacts in nearly all slices or frames are labeled as bad cases (7 and 13). Importantly, the study did not exclude patients with poor data quality, and it should be acknowledged that the feature-tracking (*FT*) based clinical software (cvi) could not provide proper strain values for 14 patients that had been neglected in the *FT* experiments.

The Deep Learning pipeline (cf. Figure 2) identifies cardiac key frames based on contraction and relaxation curves (cf. Section 5.3), as shown in previous work [32]. The derived key frame specific strain (ED-MS, MS-ED, ED-PF, PF-MD; explained in Section 5.2) enables the comparison of temporally aligned (*ED2K* and *K2K*) deformation values (cf. Figure 1 b) between patients. In this approach, strain values describe how deformation occurs towards and between key frames, making it independent of both temporal resolution and the patient-specific lengths of cardiac sub-phases. In extension to established measures such as proposed by Morales et al. [14], the strain values can be calculated in a composed (end-diastole to key frame, *ED2K*) and a novel sequential (key frame to key frame, *K2K*) manner (cf. Figure 1 b). Radial $\left(E_{rr}\right)$ and circumferential $\left(E_{cc}\right)$ strain was compared between patients with myocardial fibrosis (*LGE+*) and those without (*LGE*−). The proposed extension enables to compare strain at and between multiple key frames and segments (i.e. early diastole of AHA segment 5), thus is not limited to peak values only.

#### Statistically significant different phases and segments: Aligned Strain vs. Baseline

Consecutively, a more detailed, segment-wise analysis was conducted to identify AHA segments and corresponding phases that yield differences between *LGE+* and *LGE*− cardiac segments (cf. Figure 1 and 2 in the Supplemental Material). We compared segment-wise the strain values of *LGE+* and *LGE*− cardiac segments, i.e. key frame-specific strain (*ED2K* and *K2K*) between patients that have fibrosis in a particular segment (*LGE+*) and those that have no fibrosis in this segment (*LGE*−).

In the following Welch’s *t*-test with unpaired data and unequal variances was applied. The resulting *p*-values are adjusted for multiple testing using the Holm-Bonferroni method. The level of statistical significance was set to *p* < 0.05.

Feature-tracking based (*FT*) strain values integrated in commercial software frameworks like cvi are investigated as baseline comparison. An utilisation of all values of the whole series did not reveal statistically significant differences between *LGE+* and *LGE−* patients and segments, likely due to the temporal misalignment of the cardiac sub-phases across patients.

Within the *FT*-based peak strain values the following segments showed significant differences (adjusted p-values in brackets): $E_{rr}$ in AHA 10 (0.002), $E_{cc}$ in AHA 4,10,14 (0.042, < 0.001,0.002). *t*-Tests for the *FT* values at the predicted key frames yields the following significant phases and segments: $E_{rr}$ during ED-ES at AHA 10 (0.002), $E_{cc}$ during ED-MS at AHA 10 (0.04), $E_{cc}$ during ED-ES at AHA 4,10,14 (0.042, < 0.001,0.002).

We found that *ED*2*K* $E_{cc}$ revealed nine statistically significant differences in the AHA segments 5,6,11,12. Additionally, *ED*2*K* $E_{rr}$ showed statistical significance in the two AHA segments 5,11. Notably, ten out of eleven phases were systolic. In contrast, *K*2*K* $E_{cc}$ showed sixteen statistically significant differences in the segments 1,2,5,6,11,12,15, where eight phases were diastolic. The *K*2*K* $E_{rr}$ values additionally were significantly different at six segment-phase combinations (segments: 1,2,5,11,12), with all of them being systolic.

The *K*2*K* approach revealed not only more phases and segments being significantly different between *LGE+* and *LGE−* segments, but pointed out the importance of diastolic strain differences between healthy and fibrotic myocardium. Figure 3 b) visually captures the distribution and ranges of the *ED2K* and *K2K* strain values across all patients and AHA segments.

In summary, the *FT*-based strain curves with all values throughout the cardiac cycle did not reveal any significant segments. The *FT*-based peak strain (peak across all values) were significant in four segments, one segment for $E_{rr}$ and three for $E_{cc}$. Compared to this clinically established baseline, the DL-based aligned *ED2K* derives eleven significant differences, two $E_{rr}$ and nine $E_{cc}$ segments. The sequential *K2K* approach facilitates a phase-specific comparison, independent of previous deformations, revealing 22 significant differences — six in $E_{rr}$ and sixteen in $E_{cc}$ segments. This enhanced discriminatory capability, resulting in five times more differences, is made possible by the extraction of temporally aligned key frames.

### 2.3 *LGE*+/*−* classification with ML

Earlier works incorporating DL-based strain for abnormal deformation detection [30, 33] reported the statistic significance of their strain values in comparison with *FT*-based values or between different patient groups but they failed to show classification results, or reproducible scores. In this work strain from different segments and cardiac phases are used to evaluate the potential value of the intermediate *ED2K* and the phase specific *K2K* strain values (cf. Figure 1 b) for the task of detecting patients with myocardial fibrosis. The following experiments report the balanced accuracy, the F1-score, the sensitivity and the specificity regarding the identification of DMD patients with fibrosis. Our pipeline yields a combined strain feature vector $\vec{v}$ with $|\vec{v}|=320$ and $\vec{v}\in \left[E_{cc}/E_{rr}\times ED2K/K2K\times 5\right.$ sub-phases × 16 AHA segments]. The potential predictive values of the individual parts of $\vec{v}$ are evaluated in the following sections.

#### Validation on Development Cohort DMD57

Table 2 presents an analysis of the predictive performance of different combinations of *ED2K* and *K2K* strain values for both $E_{rr}$ and $E_{cc}$ together with the derived feature size. Machine learning models trained on a larger set of features have the potential to discover more intricate patterns for similar cases. However, they may struggle with generalization when faced with slightly different cases. Therefore, it is often crucial to identify a smaller subset of features that remains informative without becoming overly specialised. The initial part of Table 2 presents experiments incorporating both the entire set of *FT*-based strain and the corresponding peak strain baseline. The latter serves as a reference to the method proposed by Morales et al. [14], given their reported minimal discrepancies (< 1%) compared to the *FT*-based peak strain values from cvi. All metrics express the validation score as the mean ± standard deviation. The subsequent experiments are based on the strain values from our work. We begin our experiments by utilizing strain data from specific phases (cf. Section 5.2), focusing on the distinct information each phase offers. For the *ED2K* approach, we compare the following phases: early systolic (ED-MS), systolic (ED-ES), systolic + early diastolic (ED-PF), and systolic + mid diastolic (ED-MD). Notably, the ED-ES phase exhibits similarity to the traditional peak strain. In the sequential *K2K* approach, we can assess each phase independently of the preceding ones, including early systolic (ED-MS), late systolic (MS-ES), early diastolic (ES-PF), mid-diastolic (PF-MD), and late diastolic (MD-ED). Subsequent rows combine phase-specific strain values as downsampled representations of the entire cardiac cycle, and the metrics are reported for the *ED2K* and *K2K* approaches (cf. Figure 1 b). In the final rows, the *n*-th most frequently affected segments are selected, and only strain values from the significantly distinct phases observed in the DMD57 cohort (ED-MS, MS-ES, ES-PF) are incorporated.

A cross-validated grid-search (cf. Section 5.3) is applied to each of the different strain feature combinations to find a ML pipeline that generalise best for this feature-subset (cf. Table 2). On the cross-validated *DMD57* cohort most strain combinations achieved an accuracy > 70%. Within the *FT*-based experiments (first horizontal line) using all $E_{rr}$ values seem to be the best choice (acc: 75%). Unfortunately, either the sensitivity or the specificity is below 70%. Using the strain for a single cardiac phase (second horizontal line) enables an acc. > 80%. For the *ED*2*K* method strain from the early systolic phase (ED-MS) performs better than integrating further phases. The end-systolic (ED-ES) *ED2K* strain is comparable to the end-systolic strain from Morales et al. [14] and results in a similar balanced accuracy ($E_{cc}$: 83%, $E_{rr}$: 74%) on the validation splits. Incorporating the diastolic key frames yields a lower predictivity. In 75% of the cases $E_{cc}$ is more predictive and the early systolic phase (ED-MS) is more predictive than the late systolic phase (MS-ES). The most predictive single cardiac phase in the *K2K* method is the early diastolic phase (ES-PF) with a balanced acc. of 89% for the $E_{cc}$. Combining the strain values of the five key frames (frames: 5*K*) improved the training scores but performed similar on the evaluation splits. The balanced acc. of the $E_{cc}$ is greater than 80% for all 5*K* experiments. The concluding section of the table displays experiments involving tasktailored strain features, derived from segment and key frame-specific investigations detailed in Section 2.2. Only strains from cardiac phases (*ED2K*: ED-ES and *K2K*: ED-MS, MS-ES, ES-PF) demonstrating significant differences in the *DMD57* cohort are incorporated. Additionally, these experiments incorporate only the *n*-th most frequently involved segments (4*seg*,5*seg*,6*seg*,7*seg*), according to the ordered list of risk segments (AHA segment:# of involved patients): AHA5:29, AHA11:27, AHA12:23, AHA6:20, AHA4:15, AHA10:14, AHA16:14 (cf. barplot in Fig. 3 a). The robustness of the task-specific features and models becomes evident when applied to the held-out reproducibility cohort *DMD82* (cf. Table 3). Further reduction (<= 3) or increase (>= 8) in the number of incorporated segments leads to a decrease in accuracy.

#### Testing on Reproducibility Cohort *DMD82*

**Figure 4:**
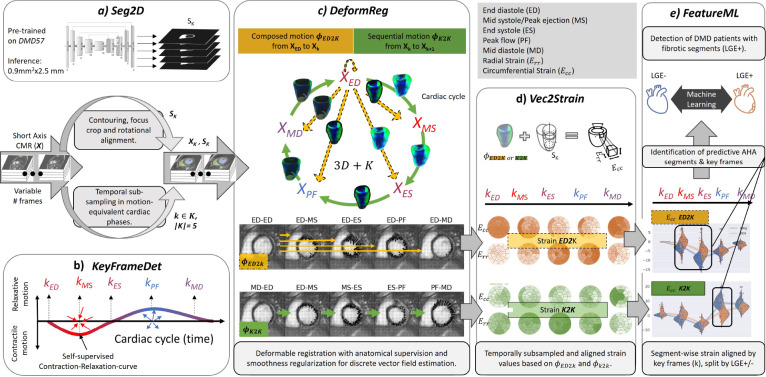
Overview of the individual modules of the Aligned strain value derivation pipeline for abnormal deformation detection (cf. Section 5.3). The modules a), b), and c) are represented by Deep Learning models. Module d) involves the calculation of Green Lagrangian strain tensors from dense displacement fields incorporating specialised basal, mid-cavity and apical polar coordinate systems, and e) encompasses a segment and phase specific strain feature exploration component along with a classical Machine Learning backbone that detects individuals with myocardial fibrosis in a cohort of patients with DMD.

The robustness is evaluated in a black-box reproducibility scenario in Table 3. Those strain combinations that resulted in a ML model with an average balanced accuracy higher than 80% were selected together with the strain experiments from the *FT*-based method to evaluate their reproducibility on new clinical data. The reproducibility cohort *DMD82* is processed by the chained and fixed pipeline of DL-models (*FrameDet ○ Seg2D ○ DeformReg*; cf. Figure 4 and Section 5.3), the corresponding feature selection approach and the fitted ML pipeline (cf. Section 5.3).

The experiments with the *FT* values dropped to a balanced accuracy < 60%. Interestingly, the strain values of the early systolic phase (ES-MS) in the *ED2K* approach are more robust (acc.: 77%) than the peak strain from ED to ES (acc.: 65%). Furthermore, from the distinct phase experiments (*K2K*) the diastolic phases ES-PF and MD-ED showed a smaller generalisation gap than the systolic phases. The early diastolic (ES-PF) *K2K* strain is still the most predictive isolated phase. Using the five key frames together (5*K*) performs +20% better than the *FT* values and +15% better than the traditional end systolic strain (ED-ES) derived from our own DL pipeline. The task-specific strain features from the last part of Table 2 results in the most robust ML models, with no performance drop between the validation splits and the test cohort (balanced acc.: +/−80%) and +30% more accurate than the *FT* baseline.

#### Relation to preserved LVEF

Further investigations into the falsely classified patients of the aligned strain and *FT*-based strain features indicated a connection between the false negative cases (LGE+ patients classified as LGE−) and the LVEF (cf. cohort characteristics in Section 2.1) as discussed below.

Deep learning based **peak systolic** $E_{cc}$ on the *DMD82* cohort (cf. row six in Table 3): 33 true positive (TP, *LGE+* classified as *LGE+*), 12 false positive (FP, *LGE*− classified as *LGE+*), 19 true negative (TN, *LGE*−, classified as *LGE*-), 18 false negative (FN, *LGE+* classified as *LGE*−). From the 18 FN cases, 13 patients have fibrotic segments but showed a normal LVEF (*LGE*+_*pLV EF*_), indicating that most FN cases are from this group and second the peak systolic strain seems not a sufficient marker to detect fibrosis in this patient group (only 52% are detected).

Deep Learning based **aligned** $E_{cc}$ **strain feature** on the *DMD82* cohort (cf. 2nd last row in Table 3): 42 TP, 6 FP, 25 TN, 9 FN and from the 9 FN cases, 8 belong to the *LGE*+_*pLV EF*_ group. Compared with the Peak systolic $E_{cc}$ strain the number of detected *LGE*+_*pLV EF*_ patients is increased by +61% (17 of 25 are detected). This indicates that the aligned $E_{cc}$ strain is potentially better in detecting fibrosis in *LGE*+_*pLV EF*_. However, 8 out of the 9 FN cases come from this group with fibrotic tissue but a preserved LVEF.

### 2.4 DL Modules

So far, the results referred to the potential value of key frame based aligned strain values. However, the validity and robustness of the intermediate modules of our pipeline (cf. Figure 2) can be underpinned by established module-specific metrics. Performance is evaluated in a stratified four-fold cross-validation manner. Four models were trained (each with 75% of the DMD57 data) per DL-task (a: semantic segmentation, b: key-frame detection and c: the deformable registration). Minor adjustments were made to *Seg2D* (cf. Figure 4 a) and section 5.3) and *KeyFrameDet* (cf. Figure 4 b) and section 5.3). Mostly, the best parameters from the original publications [32, 36] were used whenever possible to avoid dataset-driven overfitting of the models. In the Supplemental Material, the main parameters of both models are provided (cf. Supplemental Material, Model Parameters). Furthermore, on publication we will make our GitHub repository together with the detailed trainings parameter and config files publicly available.

#### Segmentation Error

We report the mean±SD Dice coefficient for the *Seg2D* module on the DMD57 cohort. To facilitate a comparison of the segmentation error we additionally evaluate the segmentation module on the publicly available ACDC [37] dataset. The 3D dice scores on the DMD57 sub-cohort are: 0.92±0.03, 0.84±0.04, 0.94±0.03, and 0.91±0.02, with a minimal dice score per 3D volume of 0.77, 0.70, 0.77, and 0.82, respectively, for the following label order: right ventricle, left ventricle myocardium, left ventricle blood pool, and all labels. Training the segmentation model with the same parameters on the publicly available ACDC cohort results in the following dice scores on the official test split: 0.90 ± 0.07, 0.86 ± 0.04, 0.92 ± 0.07, and 0.90 ± 0.03.

#### Key Frame Detection Error

With respect to the key frame detection error we report the cyclic key frame difference [32] for all five key frames on the *DMD57* dataset and for the ED and ES key frame on the ACDC dataset. In terms of key frame differences (difference in frames between the derived key frame index and the labelled key frame index), the average cyclic key frame difference on the DMD57 data is 0.81±0.77, with per key frame errors of 0.86 ± 0.77, 0.60 ± 0.75, 0.79 ± 0.67, 0.96 ± 0.76, and 1.23±1.05, corresponding to the order: end diastolic (ED), mid-systolic (MS), end-systolic (ES), peak filling (PF), and mid-diastolic (MD). The cyclic key frame difference for the ED and ES key frames on the ACDC dataset was 0.67 ± 1.05 and 1.10 ± 0.96, respectively. Figure 3 in the Supplemental Material shows the self-supervised contraction-relaxation curves that are used to derive the cardiac key frames as qualitative indicator for the *DMD82* cohort where no key frame labels exists and no key frame detection error can be calculated. The line-plot shows one curve per patient that describes the dynamic contraction/relaxation behaviour throughout the cardiac cycle. Values below 0 refers to frames with a contracting motion pattern and values above to relaxing frames.

#### Registration Error

The quantification of the deformable registration module *DeformReg* and the anatomical plausibility of the learnt displacement field $\phi_{n}$ involves reporting the Dice coefficient and Hausdorff Distance between all $s_{f}$ and $s_{k}\circ \phi_{n}$ (cf. Section 5.3) volume pairs (please note that $s_{f}$ and $s_{k}$ are considered as a weak ground truth). The mean Dice and Hausdorff Distance across all patients and key frame pairs $s_{k}\circ \phi_{k2k}$ and $s_{k+1}$ is 0.827± 0.038, 8.467 ± 6.747mm. The detailed error per key frame is given in Table 6 in the Supplemental Material.

## 3 Discussion

Most non-contrast models do not meet thresholds for routine clinical use such that model performance remains suboptimal [13]. The fully automated pipeline (cf. Figures 2, 4 and Section 5.3) presented in this study excels at generating aligned *ED2K* and *K2K* strain values (cf. Figure 1) that demonstrate superior predictive capabilities (cf. Table 2 and 3) when compared to strain values obtained using recent *FT*-based clinical software or recent unaligned Deep Learning methods [30]. The authors in [30] state a disagreement < 1% compared to the circumferential end systolic strain values from the feature tracking approach (*FT*) implemented in Circle cvi42 (cvi), indicating similar predictive capabilities.

The alignment of strain values in our approach enables detailed comparisons (cf. Table 3 and Figure 2 in the Supplemental Material) on a segment-by-segment and cardiac phase basis, leading to the identification of five times more significant differences in strain between patient groups. The introduction of novel sequential (*K2K*) strain values (cf. Figure 1 and Sections 5.3 and 5.3) complements the approach, particularly during diastolic phases of interest in patients with myocardial scar but preserved LVEF (cf. significant segments in Table 3 in the Supplemental Material). The study found that sequential strain values provided a higher frequency of statistically significant differences between patient groups compared to traditional composed (*ED2K*) strain values, especially during cardiac relaxation (significant segments: *FT* peak strain (4) vs *ED2K* (11) vs K2K (22)).

Leveraging the insights from the comparative analysis in Section 2.2, the authors developed a tasktailored and resilient strain feature set based on the insights from the *DMD57* cohort in Section 2.3 capable of distinguishing between patients with and without myocardial fibrosis using standard CMR data alone. This approach was cross-validated on 57 patients with a sensitivity and specificity of 0.83±0.11, 0.87 ± 0.08 and after model development black-box tested on the second held-out cohort (*DMD82*) with similar performance of 0.81 and 0.84 (cf. Tables 2 and 3). This pure inference scenario on a completely separated test set ensures that insights into the generalisation capability are given without potential bias introduced by just splitting a single cohort into train and test, which often leads to overoptimistic results due to model optimisation. Both cohorts consists of challenging CMR cases but no case was excluded in our pipeline (in total 20 bad cases and 70 cases of medium quality); 14 of these cases could not even be processed with a clinical software and had to be excluded in the results for the state-of-the art feature tracking method.

A significant performance drop could be observed when performing an ablation experiment in the temporal domain. We limited the *ED2K* strain computation to end-systolic strain and showed that only four segments with significant differences could be identified (ED to ES - cf. Table 2 and 3 in the supplemental Material) with a balanced accuracy of 65% on the *DMD82* cohort (cf. row 6 in Table 3). Furthermore, the peak systolic $E_{cc}$ was only able to detect half of the *LGE*+_*pLV EF*_ patients (cf. Section 2.3). In contrast, the aligned $E_{cc}$ strain resulted in 50% less FN cases and was able to identify +61% of the *LGE*+_*pLV EF*_. The improved predictive capabilities of aligned strain to detect patients with preserved LVEF and maybe less ‘abnormal’ systolic deformations highlights the advantage of integrating several aligned temporal strain values into the decision-making process. The superior performance potentially results from the fact that the same phenomenon (abnormal deformation) can be observed in multiple phases leading to a more stable classification, and/or the information on (subtle) changes in deformation only evident in single, potentially nonendsystolic phases, is now integrated. In contrast to many prior work [14, 33, 24, 38, 29], strain values at various cardiac phases are automatically detected through a self-supervised approach [32] for further processing without the necessity of prior handpicking of a specific phase. This makes the approach more flexible and general, as different diseases certainly require tailored combinations of strain features to reach a high level of accuracy.

Segmentation and key frame detection errors have an impact on the overall performance of the classifier. The biventricular segmentation model usually performs better on ED frames [37], which is an additional challenge for the sequential strain computation (*K2K*) as it requires the creation of new AHA segment masks at each key frame. However, in our single experiments we could show that the approaches are robust (cf. Section 2.4) and especially the choice of a self-supervised approach is powerful with respect to translation to other cohorts where no temporal labels are given.

In this study we split the patients with DMD into binary groups of patients with *LGE+* segments and without. This does neither reflect the amount of fibrosis per segment nor the total amount of fibrotic tissue on the myocardium. For treatment planning it is primary important if there is fibrotic tissue at all, which is reflected in our design. More detailed failing case analysis revealed that 8 out of the 9 falsely negative predicted cases from the aligned strain approach (Table 3, 2nd last row) belong to the group of *LGE*+_*pLV EF*_, questioning the relationship of late gadolinium deposition and abnormal strain in some patients. Patients with minimal late gadolinium deposition in only a few cardiac segments, despite their involvement, may not necessarily display an ‘abnormal’ strain pattern, backing up the argument that strain is an interesting feature itself and that most of our false predictions can be explained, increasing trust into the approach.

Multiple studies have previously investigated areas of positive LGE in patients with DMD. Reported LGE patterns in DMD include LV inferolateral (AHA5,11), anterolateral (AHA6,12), inferior (AHA4,10) and other free wall segments [30, 37]. Hor et al. investigated the relationship among LGE, age and LVEF in a cohort of 314 patients with DMD. LGE was present at a higher rate in the free wall compared to the septal wall (42.7% versus 5.3%). At base and mid-cavity, anterolateral, inferolateral, and inferior segments had the highest counts of LGE positivity (n=190,165,54) [39]. According to Siegel et al., segments in DMD known not to develop LGE are located inferoseptal and anteroseptal [40]. Figure 3 a confirms the localisation of *LGE+* segments in general. Furthermore, this work extends the localisation by temporal information regarding the cardiac phases that are affected per segment. Hor et al. reported in a cohort of 70 patients with DMD and 16 controls that global mean circumferential strain decreased with advancing age. However, in this study, only the midventricular slice was investigated and peak strain was referred to as ED-ES strain [38]. Also, in a cohort of 68 patients with DMD and 15 controls it was shown that LV free wall has the greatest decline of circumferential strain in older patients with DMD. There, the lateral free wall midcircumferential segments exhibited the greatest declines in circumferential strain [41]. Similarly, Siegel et al. reported in a cohort of 24 patients with DMD and eight controls that CMR-Feature Tracking average $E_{cc}$ was reduced in the anterolateral, inferolateral and inferior segments [40]. Raucci et al. [13] performed strain analysis via feature tracking in 66 patients to predict presence of LGE in patients with DMD. The model performance to predict presence of LGE yielded an AUC of 0.74 and, a highest balanced accuracy of 71.8% with a sensitivity of 74.3% and specificity of 69.2%. Siddiqui et al. found in a cohort of 30 patients with DMD with and without cardiomyopathy that the 3D global $E_{cc}$ was the strongest predictive marker for DMD-associated cardiomyopathy [42]. The segment-wise analysis conducted in this work (cf. Table 3 in the Supplemental Material) highlights the predictiveness of the free wall segments (5, 6, 11 and 12, 15 and 16). Considering that the segments 5 and 11 are affected in more than 96% of the *LGE+* patients, it may be helpful to focus on those for a patient-based *LGE+* classification.

## 4 Conclusion

The proposed novel fully-automatic Deep Learning pipeline excels at generating aligned strain values for cardiac sub-phases and exhibit superior predictive capabilities compared to traditional strain values (+30% acc.) from variable CMR scans. The alignment of the strain values empowers detailed strain comparisons (detecting five times more significant differences) on a segment-by-segment and cardiac phase basis, which is not possible with existing strain measures. In the task of myocardial fibrosis detection, a sensitivity and specificity > 80% is achieved on a blinded reproducibility cohort of 82 patients with DMD. This approach may reduce the necessity of repeated gadolinium-based contrast administration and contribute to the earlier identification of cardiac fibrosis in patients with DMD.

## 5 Methods

### 5.1 Magnetic Resonance Imaging Acquisition Parameters

In both cohorts, all but two scans have been performed on a Philips Healthcare Ingenia scanner at 1.5T (Philips Healthcare, Best, Netherlands). The CMR images were captured in stacks of 2D cineSSFP short-axis sequences. Table 1 in the Supplemental Material summarises the imaging parameters. The scans capture 30 frames, the sequence lengths (ms) are in a range of [390,930] (*DMD57*) and [390,1080] (*DMD82*), which represent a heart rate between 55bpm and 153bpm. The LGE acquisition (cf. Section 5.2) was performed on the same MR scanner at mid-diastole.

### 5.2 Clinical Annotations

#### Expert Labels

A pediatric cardiologist with at least five years of experience labelled five key frames *K* in the *DMD57* cohort. Key frame annotations consist of end-diastole (ED, end of diastolic relaxation); mid-systole (MS, maximum systolic contraction); end-systole (ES, end of systolic contraction); peak flow (PF, peak early diastolic relaxation) and mid-diastole (MD, key frame before atrial contraction at p-wave onset). Manual contouring of the right ventricle (RV), left ventricle (LV) and the myocardium (LVMYO) was conducted by the same pediatric cardiologist on the five key frames of the *DMD57* cohort. Standard contouring techniques were followed [43] and clinically established software was used (Circle cvi42 version release 5.11.2, Circle Cardiovascular Imaging Inc., Calgary, Alberta, Canada).

#### Manual derivation of LGE diagnosis

The patients underwent LGE examination (Phase Sensitive Inversion Recovery, PSIR). The administered contrast agent was GADOVIST (1.5 mmol per kg body weight). The developed approach does not rely on any LGE measurements during training or prediction. The LGE ground truth was provided on a segmental level for each patient and was extracted from a routine clinical report by a pediatric cardiologist with at least five years of experience. The decision of segmental classification as either LGE+ or LGE− was then visually confirmed by a second independent observer for the purpose of this study. Individuals with at least one LGE+ cardiac segment were classified as LGE+. This approach is explained by current clinical decision making during treatment planning, where the primary important factor is the occurence or absence of a LGE+ segment.

### 5.3 Model definition

An automatic pipeline is proposed (cf. Fig. 4) with the following modules.

In this study, we combine two existing models: *Seg2D* [44] for spatial grouping (cf. Section 5.3) and the *KeyFrameDet* model [32] for temporal alignment (cf. Section 5.3). Additionally, we propose a deformable registration model (cf. *DeformReg* in Section 5.3) to predict two sets of temporally aligned 3D+t displacement fields from cine CMR sequences. One displacement field describes composite (*ED2K*) motion from end-diastole (ED) to predefined cardiac key frames, while the other represents sequential motion (*K2K*) from one key frame to the next (cf. Figure 1). We extend Morales et al.’s Green Lagrangian strain calculation method [14] to compute sequential myocardial strain with varying polar coordinate systems per apical, mid-cavity and basal area (35%, 35%, and 30% as suggested in [26]) from the displacement fields (cf. Section 5.3). Finally, we evaluate the strain values for myocardial scar detection using cross-validated grid search with approximately 2000 different machine-Learning methods/parameters.

#### DL-based Segmentation

Module a) in Figure 4 refers to a 2D supervised biventricular segmentation model (*Seg2D*). The short-axis contours (cf. Section 5.2) are used to train the model in predicting bi-ventricular masks for anatomical regularisation in *DeformReg* (cf. Section 5.3) and for spatial assignment of the strain values (cf. Section 5.3) according to the 16 myocardial AHA segments (*Vec2Strain*) [26]. This model has a modified U-Net architecture [45] and was reused from earlier works [36, 44, 46], still achieving state-of-the-art mean Dice scores (cf. Section 2.4) without any specific modifications.

#### DL-based Key Frame Detection

The self-supervised cardiac key frame detection model (*KeyFrameDet*) in Figure 4 b), introduced by Koehler et al. [32] is employed to derive cardiac key frames (*K*) (cf. Section 5.2), based on a global contraction-relaxation curve from a stack of short-axis CMR sequences. These key frames are used to divide a cardiac cycle into cardiac subsequences. This enables to learn key frame specific discrete displacement fields and by this derive intermediate strain values (ED-MS: early systolic, MS-ES: late systolic, ES-PF: early diastolic, PF-MD: mid-diastolic and MD-ED: late diastolic) that describe the same cardiac phases across patients. In Section 2.4 we report the cyclic frame differences [32] between those predictions and the ground truth. It should be noted that the approach does not require expert annotation for training and can therefore be easily trained on various CMR cohorts as demonstrated in [32].

#### DL-based Deformable Registration

**Figure 5:**
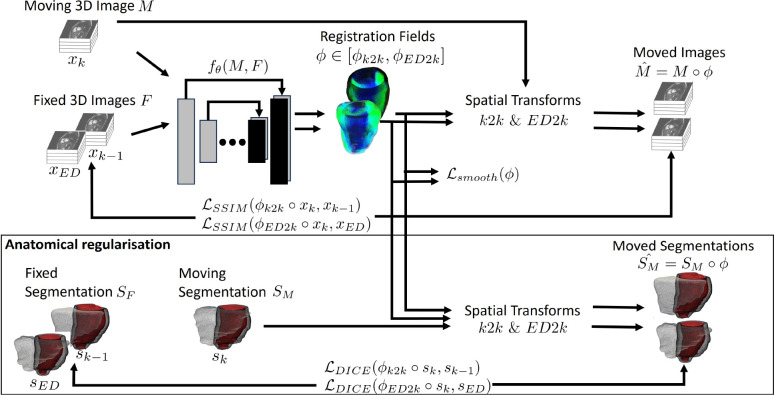
DeformReg is designed to learn and predict both short- and long-distance displacement fields, denoted as ϕ, while incorporating anatomical guidance.

After temporal and spatial alignment c) refers to a 3*D* + *K* deformable registration model (*DeformReg* - cf. Figure 5) that learns to predict a discrete composed (*ED2K*) and sequential (*K2K*) displacement field ϕ (cf. Figure 1) for the five cardiac subsequences, sliced by K. The backbone is a 3D U-Net [45] followed by several bilinear spatial transformer layers originally introduced by Jaderberg et al. [47] The model is trained jointly on five 3D CMR volumes and masks, with the masks generated by *Seg2D*. This model produces two displacement fields: one for composed $\phi_{ED2k}$ and one for sequential $\phi_{k2k}$ displacement. These fields utilize grey value registration and anatomical guidance via the myocardium mask, demonstrating the motion in similar cardiac phases among patients. For this model, a 3D+K stack of CMR sequences is defined as X, where each 3D CMR volume is denoted as x∈X, and the corresponding 3D myocardial mask is denoted as s∈S, where $x_{k}$ and $s_{k}$ refer to a 3D CMR/ mask at a specific cardiac key frame K∈[ED,MS,ES,PF,MD].

During training, the general registration task of ϕ, $\overset{ˆ}{M}=f_{\theta }(M,F)$ is addressed, with M and F representing the moving and fixed volume pair, respectively. θ represents the learnable parameters of f, ϕ represents the learned discrete displacement field, and $\overset{ˆ}{M}$ represents the moved volume after the spatial transformer layer applies ϕ to M. Figure 5 provides a visual description of the registration process. One forward pass of *DeformReg* processes the whole 3D+k volume X simultaneously.

This model processes X in one forward pass, and creates temporally consecutive moving $\left(x_{k}\right)$ and fixed $\left(x_{k-1}\right)$ volume pairs. Figure 5 shows the general architecture. Two vector fields ϕ are learned. The first one is $\phi_{k2ED}$, which describes the composed deformation from $x_{k}$ (moving) to $x_{ED}$ (fixed). The second one is $\phi_{k2k}$, which reflects the sequential deformation from $x_{k}$ (moving) to the previous $x_{k-1}$ (fixed). To ensure physiological plausibility, both deformation fields $\phi_{k2k}$ and $\phi_{k2ED}$ are additionally applied to the corresponding myocardial mask $s_{k}$. The bilinear spatial transformer layers samples grey values from the target grid $x_{ED}$ or $x_{k-1}$, based on the discrete vector fields $\phi_{k2ED}$ and $\phi_{k2k}$ and solve the deformable registration task of the moving and fixed volume pairs. The learnt displacement fields ϕ are later treated as the voxel-wise ’forward‘ displacement or motion over time. The learning procedure comes from the three registration losses:

(1) $$ℒ\left(F,M,\phi_{k2k}\right)=\lambda_{k2k}{ℒ}_{SSIM}(\left.x_{k-1},x_{k}\circ \phi_{k2k}\right)\;\;+\lambda_{\text{reg}}{ℒ}_{s}\left(\phi_{k2k}\right)$$

(2) $$ℒ\left(F,M,\phi_{k2ED}\right)=\lambda_{k2ED}{ℒ}_{SSIM}\left(x_{ED},x_{k}\circ \phi_{k2ED}\right)\;\;+\lambda_{reg}{ℒ}_{s}\left(\phi_{k2ED}\right)$$

(3) $$ℒ\left(F,M,\phi_{n}\right)=\lambda_{DICE}{ℒ}_{DICE}\left(s_{F},s_{M}\circ \phi_{n}\right)\;\;+\lambda_{reg}{ℒ}_{s}\left(\phi_{n}\right)$$,

where n belongs to the set [k2ED,k2k]. $S_{F}$ and $S_{M}$ are the concatenated fixed and moving mask pairs associated to the composed and sequential displacement fields $\phi_{k2ED}$ and $\phi_{k2k}$. Each image similarity loss component and its corresponding regulariser are assigned unique weights to control their influence on the learning process, denoted as $\lambda_{k2ED}$, $\lambda_{k2k}$, $\lambda_{DICE}$, and $\lambda_{reg}$. These losses propagate their gradients back into the same encoder and optimise simultaneously to account for both short- and long-distance deformations while adhering to anatomical structure and smoothness constraints. A consistent smoothness regulariser ${ℒ}_{s}(\phi )$ is part of the three registration losses:

(4) $${ℒ}_{s}(\phi )=\sum p\in \Omega ∥\nabla \phi (p){∥}^{2}.$$

This loss component uses the spatial gradient of eight neighbouring voxels to enforce local smoothness of the displacement field, following the approach introduced by Balakrishnan et al. [48].

In response to the challenge of precise registration of myocardial tissue and its boundaries caused by variations in image brightness, we incorporate a modified Structural Similarity Index measure (SSIM) as robust image similarity loss component. This loss function assesses luminance, contrast, and structural features for the two fixed and moved volume pairs: $\left(x_{ED},x_{k}\circ \phi_{k2ED}\right)$, $\left(x_{k-1},x_{k}\circ \phi_{k2k}\right)$. The original SSIM measure is only defined for 2D images. Here, ${ℒ}_{SSIM}$ is defined as $\frac{1}{\left|z\right|}\times \sum_{0}^{\left|z\right|}\;{ℒ}_{SSIM,slice}$, with z∈Z referring to each slice in $x_{k}$. The image similarity loss ${ℒ}_{SSIM}$ for one fixed and moving slice is defined as:

(5) $$\begin{array}{l} {ℒ}_{SSIM,slice}\left(f,m\circ \phi_{k2k}\right)=\\ \;\frac{\left(2\mu_{f}\mu_{m}\;+\;\epsilon_{1}\right)\left(2\sigma_{fm}\;+\;\epsilon_{2}\right)}{\left(\mu_{f}^{2}\;+\;\mu_{m}^{2}\;+\;\epsilon_{1})(\sigma_{f}^{2}\;+\;\sigma_{m}^{2}\;+\;\epsilon_{2}\right)}. \end{array}$$

Here, f and m refer to one fixed and moving slice pair in $x_{k-1}$ and $x_{k}$, $\mu_{f}$ and $\mu_{m}$ represent the average, while $\sigma_{f}^{2}$ and $\sigma_{m}^{2}$ represent the variance of a N×N region in f and m. $\sigma_{fm}$ is the co-variance of f and m, and $\epsilon_{1}$ and $\epsilon_{2}$ are variables introduced to prevent instability. The parameters are chosen as defined in the original paper [49]. The same image similarity measure is used to learn the traditional composed displacement field $\phi_{k2ED}$ in Eq. 2.

For anatomical regularisation of the deformation fields, a DICE component, as introduced by Milletari et al. [50], is employed as image similarity measure for the myocardial mask $s_{k}$ and the moving counterpart $s_{k-1}\circ \phi_{k2k}$:

(6) $$\begin{array}{l} {ℒ}_{DICE}\left(s_{k},s_{k-1}\circ \phi_{k2k}\right)=\\ \;1-2\times \frac{\left|s_{k}\;\cap \;\left(s_{k-1}\;\circ \;\phi_{k2k}\right)\right|\;+\;\epsilon }{\left(\left|s_{k}\right|\;+\;\left|s_{k-1}\;\circ \;\phi_{k2k}\right|\right)\;+\;\epsilon },\; \end{array}$$

where ϵ=1 is included for smoothing purposes.

#### Vector2strain

Followed by d) (cf. Figure 4), a **Vector2strain** module (Vec2Strain) that interprets $\phi_{k2k}$ and $\phi_{k2ED}$ and derives the voxel-wise radial and circumferential strain $\left(E_{rr}/E_{cc}\right)$ over time. $E_{rr}$ and $E_{cc}$ values are then assigned and averaged for the 16 AHA segments. This results in 16 segmental strain values for each *ED2K* and *K2K* strain measurement, each of which is temporally aligned due to K across patients. Morales et al. [14] proposed a solution to calculate the Green Lagrangian strain tensors from a displacement field. The *Vec2Strain* module is an extension to the existing approach that enables to derive sequential (*K2K*) strain values.

#### ML-based Fibrosis Detection

Finally, e) in Figure 4 refers to a **feature selection and evaluation** module (*FeatureML*) that identifies predictive segments at cardiac phases for the task of detecting patients with myocardial fibrosis (LGE+ cf. Section 5.2). In order to quantify spatially mapped myocardial deformation at and between different cardiac key frames, the myocardium is divided into 16 segments, according to the definition of the American Heart Association (AHA) [26] and leaving out the most apical segment AHA17. Temporally down-sampled and aligned $E_{rr}$ and $E_{cc}$ values are acquired from the *Vec2Strain* module for $\phi_{k2ED}$ and $\phi_{k2k}$. In summary, we can derive 320 strain values. In section 2.3 we report the results for various strain value combinations. During training, *FeatureML* identifies AHA segments at cardiac phases that show significantly different strain values $v_{n}$ between LGE+/LGE− patients in the training subset.

A grid-search through more than 2000 different ML pipelines was performed to find a parameter combination that shows the best generalisation capabilities for each of the selected strain combinations. During inference, this module applies the same feature extraction and forwards the strain values for the segments and phases of interest to the trained ML model in order to get a binary classification as either *LGE+* or *LGE−*. After the paper’s acceptance, our GitHub repository, along with the experiment and model configuration files and detailed parameters, will be made publicly available.

## Figures and Tables

**Table 1:** Characteristics of the two DMD cohorts.

	DMD57 (*LGE+* vs LGE-)			DMD82 (*LGE+* vs LGE-)			57 vs 82

Parameter	*LGE+*	*LGE*-	*p*-value	*LGE+*	*LGE*-	*p*-value	*p*-value

N	33	24	n/a	51	31	n/a	n/a
Age (yrs)	15.2 ± 3.1	12.8 ± 2.7	0.002	n/a	n/a	n/a	n/a
Weight (kg)	62.7 ± 24.5	48.0 ± 14.9	0.007	61.2 ± 27.9	49.4 ± 19.1	0.025	0.958
Height (cm)	150.5 ± 15.9	147.0 ± 15.9	0.403	144.0 ± 19.7	142.9 ± 19.7	0.792	0.073
BSA (m^2^)	1.6 ± 0.4	1.4 ± 0.3	0.009	n/a	n/a	n/a	n/a
HR (bpm)	93.5 ± 14.5	97.2 ± 15.5	0.353	n/a	n/a	n/a	n/a
BP (sys,mmHg)	114.2 ± 13.4	110.3 ± 10.3	0.229	n/a	n/a	n/a	n/a
BP (dia,mmHg)	72.0 ± 10.7	72.3 ± 10.2	0.926	n/a	n/a	n/a	n/a
LVEF (%)	51.5 ± 9.0	58.4 ± 7.2	0.002	52.9 ± 9.2	59.1 ± 3.9	< 0.001	0.583
LVEDV (mL)	122.9 ± 44.2	89.3 ± 18.0	< 0.001	115.7 ± 61.2	96.2 ± 37.5	0.077	0.952
LVESV (mL)	61.9 ± 32.1	37.7 ± 11.6	< 0.001	58.6 ± 53.6	39.7 ± 16.9	0.023	0.965
LV mass (g)	76.3 ± 30.6	55.2 ± 11.5	0.001	65.0 ± 25.7	59.1 ± 22.2	0.279	0.294

Values are stated as mean±SD. *p*-values were calculated with two-sample *t*-tests and corrected for multiple testing between the *LGE+* and *LGE*- groups within the DMD57 (*3rd* column) and DMD82 (6*th* column) cohorts. The last column compares both cohorts independent of the LGE class. BSA = body surface area, HR = heart rate, BP = blood pressure (systolic,diastolic), LVEF = left-ventricular ejection fraction, LVEDV = left-ventricular end-diastolic volume, LVESV =left-ventricular end-systolic volume.

**Table 2:** Binary classification of patients as *LGE+* or *LGE*- mean values for the cross-validation splits of the *DMD57* cohort

Method	Frames	E	#Features	Mean±SD validation measures for the four folds of *DMD57_cv_*			
				Balanced Acc. ↑	F1-score ↑	Sensitivity ↑	Specificity ↑

*FT*	all	$E_{cc}$	480	.69 ± .15*	.70 ± .16	.63 ± .15	.71 ± .27
*FT*	all	$E_{rr}$	480	.75 ± .07*	.74 ± .05	.66 ± .10	.84 ± .20
*FT*	peak	$E_{cc}$	16	.66 ± .14*	.75 ± .07	.78 ± .08	.54 ± .30
*FT*	peak	$E_{rr}$	16	.65 ± .13*	.57 ± .19	.46 ± .18	.83 ± .15

*ED*2*K*	ED-MS	$E_{cc}$	16	.83 ± .10	.84 ± .08	.81 ± .12	.84 ± .20
*ED*2*K*	ED-MS	$E_{rr}$	16	**.84** ± .08	**.87** ± .07	**.89** ± .11	**.80** ± .13
*ED*2*K*	ED-ES	$E_{cc}$	16	.83 ± .15	.84 ± .12	.79 ± .08	.88 ± .24
*ED*2*K*	ED-ES	$E_{rr}$	16	.74 ± .13	.77 ± .12	.79 ± .15	.68 ± .20
*ED*2*K*	ED-PF	$E_{cc}$	16	.74 ± .13	.74 ± .16	.69 ± .21	.79 ± .13
*ED*2*K*	ED-PF	$E_{rr}$	16	.66 ± .23	.76 ± .18	.83 ± .21	.50 ± .37
*ED*2*K*	ED-MD	$E_{cc}$	16	.70 ± .10	.78 ± .08	.85 ± .09	.54 ± .15
*ED*2*K*	ED-MD	$E_{rr}$	16	.61 ± .12	.70 ± .10	.76 ± .15	.46 ± .15

*K*2*K*	ED-MS	$E_{cc}$	16	.81 ± .10	.83 ± .09	.81 ± .12	.80 ± .18
*K*2*K*	ED-MS	$E_{rr}$	16	.80 ± .04	.84 ± .02	.89 ± .02	.72 ± .16
*K*2*K*	MS-ES	$E_{cc}$	16	.75 ± .09	.81 ± .09	.85 ± .14	.66 ± .12
*K*2*K*	MS-ES	$E_{rr}$	16	.72 ± .12	.74 ± .12	.73 ± .16	.71 ± .20
*K*2*K*	ES-PF	$E_{cc}$	16	**.89** ± .06	**.89** ± .07	**.82** ± .15	**.96** ± .08
*K*2*K*	ES-PF	$E_{rr}$	16	.63 ± .12	.76 ± .07	.88 ± .06	.38 ± .27
*K*2*K*	PF-MD	$E_{cc}$	16	.71 ± .03	.75 ± .05	.73 ± .11	.70 ± .13
*K*2*K*	PF-MD	$E_{rr}$	16	.59 ± .08	.60 ± .11	.55 ± .12	.63 ± .06
*K*2*K*	MD-ED	$E_{cc}$	16	.80 ± .13	.78 ± .15	.73 ± .22	.88 ± .10
*K*2*K*	MD-ED	$E_{rr}$	16	.60 ± .07	.67 ± .05	.71 ± .20	.50 ± .32

*ED*2*K*	5*K*	$E_{cc}$	64	.84 ± .11	.86 ± .09	.85 ± .09	.84 ± .15
*ED*2*K*	5*K*	$E_{rr}$	64	.79 ± .10	.82 ± .07	.82 ± .11	.76 ± .23
*K*2*K*	5*K*	$E_{cc}$	80	.80 ± .13	.78 ± .15	.73 ± .22	.88 ± .10
*K*2*K*	5*K*	$E_{rr}$	80	.72 ± .09	.80 ± .05	.89 ± .11	.55 ± .29
*K*2*K*, *ED*2*K*	5*K*	$E_{cc}$	144	.82 ± .12	.81 ± .13	.76 ± .20	.88 ± .10
*K*2*K*, *ED*2*K*	5*K*	$E_{rr}$	144	.81 ± .11	.86 ± .08	.91 ± .07	.71 ± .20

*K*2*K*,*ED*2*K*	3*K*, 4*seg*	$E_{cc}$	16	.82 ± .07	.84 ± .05	.84 ± .11	.80 ± .18
*K*2*K*, *ED*2*K*	3*K*, 5*seg*	$E_{cc}$	20	**.83** ± .11	**.87** ± .08	**.94** ± .11	**.72** ± .20
*K*2*K*, *ED*2*K*	3*K*, 6*seg*	$E_{cc}$	24	.81 ± .08	.84 ± .05	.85 ± .13	.76 ± .23
*K*2*K*, *ED*2*K*	3*K*, 7*seg*	$E_{cc}$	28	.79 ± .09	.81 ± .07	.79 ± .12	.79 ± .22

*FT*: feature tracking baseline approach from the Circle cvi42 software, *ED*2*K*: End-diastole to key frames (aligned composed), *K*2*K*: key frame to key frame (aligned sequential), ED:end-systole, MS:mid-systole, ES:end-systole, PF:peak-flow,MD:mid-diastole, 5K:include five key frames, 3K,n-seg: include ED-MS,MS-ES,ES-PF and the *n* most frequent segments in *DMD57* (cf. Fig. 3), $E_{rr}$: radial strain, $E_{cc}$: circumferential strain. Up-arrows indicate increasing quality at higher values.

* For four patients of *DMD57* cohort the *FT*-based method was not able to derive strain values, thus these patients are excluded in the corresponding experiments.

**Table 3:** Reproducibility - Binary classification of patients as *LGE+* or *LGE*- for the held-out *DMD82* cohort

Method	Frames	E	#Features	Test measures for the held-out *DMD82*			
				Balanced Acc. ↑	F1-score ↑	Sensitivity ↑	Specificity ↑

*FT*	all	$E_{cc}$	480	.58*	.73	.82	.33
*FT*	all	$E_{rr}$	480	.49*	.71	.84	.15
*FT*	peak	$E_{cc}$	16	.51*	.74	.91	.11
*FT*	peak	$E_{rr}$	16	.55*	.37	.25	.85

*ED*2*K*	ED-MS	$E_{cc}$	16	.77	.76	.66	.87
*ED*2*K*	ED-ES	$E_{cc}$	16	.65	.69	.65	.65
*ED*2*K*	ED-MS	$E_{rr}$	16	.63	.67	.61	.65
*K*2*K*	ED-MS	$E_{cc}$	16	.67	.69	.61	.74
*K*2*K*	ES-PF	$E_{cc}$	16	.78	.75	.63	.93
*K*2*K*	MD-ED	$E_{cc}$	16	.77	.76	.67	.87
*K*2*K*	ED-MS	$E_{rr}$	16	.63	.67	.61	.65

*ED*2*K*	5*K*	$E_{cc}$	64	.61	.71	.71	.52
*K*2*K*	5*K*	$E_{cc}$	80	.77	.76	.67	.87
*K*2*K*, *ED*2*K*	5*K*	$E_{cc}$	144	.73	.75	.69	.77
*K*2*K*, *ED*2*K*	5*K*	$E_{rr}$	144	.70	.74	.69	.71

*K*2*K*, *ED*2*K*	3*K*, 4*seg*	$E_{cc}$	16	.75	.76	.66	.84
**K2K,ED2K**	**3K,5seg**	$E_{cc}$	20	**.81**	**.84**	**.80**	**.81**
**K2K,ED2K**	**3K,6seg**	$E_{cc}$	24	**.81**	**.85**	**.82**	**.81**

Applying the methods from Table 2 that results in balanced accuracy > 80%, without further modifications, on the *DMD82* to estimate the robustness of various strain combinations. *FT*: feature tracking baseline approach from the Circle cvi42 software, *ED2K*: End-diastole to key frames (aligned composed), *K2K*: key frame to key frame (aligned sequential), ED:end-systole, MS:mid-systole, ES:end-systole, PF:peak-flow, MD:mid-diastole, 5K:include five key frames, 3K,*n*-seg: include ED-MS,MS-ES,ES-PF and the *n* most frequent segments in *DMD57* (cf. Fig. 3), $E_{rr}$: radial strain, $E_{cc}$: circumferential strain. Up-arrows indicate increasing quality at higher values.

* For ten patients of *DMD82* cohort the *FT* method was not able to derive strain values, thus these patients are excluded in the corresponding experiments.

## Data Availability

The datasets used and/or analysed during the current study are available from the corresponding author on reasonable request and with appropriate data use agreement. Furthermore, on publication we will make our GitHub repository together with the detailed trainings parameter and config files publicly available.
